# Hemodynamic Responses to Simulated Long Working Hours with Short and Long Breaks in Healthy Men

**DOI:** 10.1038/s41598-018-32908-y

**Published:** 2018-09-28

**Authors:** Xinxin Liu, Hiroki Ikeda, Fuyuki Oyama, Keiko Wakisaka, Masaya Takahashi

**Affiliations:** 1 National Institute of Occupational Safety and Health, Japan, Nagao 6-21-1, Tama-ku, Kawasaki, Kanagawa 214-8585 Japan; 2 Department of Design Science Graduate School of Engineering, Chiba University, Yayoi 1-33, Inage-ku, Chiba, 263-8522 Japan

## Abstract

This study aimed to examine hemodynamic responses and the necessity of breaks under long working hours. Thirty-eight healthy males conducted PC-based work from 9:10 to 22:00. Nine 10-minute short breaks and two long breaks (a 1-hour break and a 50-minute break) were provided, and hemodynamic responses were measured regularly during this period. The results showed that systolic blood pressure increased during the working hours and cardiovascular burden increased under long working hours. Cardiac responses decreased, but vascular responses increased continually during work periods without long breaks. The long breaks, however, benefitted workers by preventing excessive decreases in cardiac responses and increases in vascular responses, but this effect may decrease with the extension of working hours. In conclusion, long working hours increase cardiovascular burden, and taking long breaks is important for reducing these burdens when long working hours cannot be avoided.

## Introduction

Long working hours are considered to be associated with increases in various health problems. The compensatory control model reported by Hockey^1^ suggested that work performance under stress may be protected by increasing subjective effort and behavioral and physiological reactions. To sustain work performance during prolonged working hours, more resources are needed, and this results in increased physiological and psychological reactions. On the other hand, the effort-recovery model reported by Meijman and Mulders^2^ indicated that negative effects on health by work occur under continued exposure to workload without sufficient recovery of increased physiological and psychological activities. Generally, long working hours directly connect with a heavy workload, poor sleep, and insufficient recovery, and all these factors are directly or indirectly related to an increased risk of various diseases, especially cardiovascular diseases. Published systematic reviews and cohort studies have reported that cardiovascular morbidity and mortality significantly increase if working hours exceed 50–55 hours per week, compared with standard hours (35–40 hours per week)^3–5^. In Asia, long working hours have become a major social issue in recent years. For example, 260 Japanese workers’ cerebrovascular and cardiovascular diseases were considered to be caused by overworking in 2016, and 90% of these workers worked over 60 hours per week^6^. However, approximately 4.3 million workers still work more than 60 hours per week (80 hours overtime per month on average) in Japan alone^7^, and protecting these workers from overwork-related diseases is an emergency issue.

Previous studies have suggested that cardiovascular measures are indicated in terms of different work-related strain outcomes. For example, Vrijkotte *et al*. reported that high work stress, which is defined as a combination of high effort and low reward at work, was associated with a higher heart rate (HR) and a higher systolic blood pressure (SBP) during work^8^. In addition, cardiovascular responses are also associated with cognitive and emotional state at work, and negative appraisal of the work and negative emotional state at work were associated with stronger blood pressure (BP) reactivity^9,10^. Therefore, elucidation of the cardiovascular response is important for evaluating the work-related burden under long working hours. BP levels during standard working hours (generally 8 hours per day) are known to be higher than during other times, such as while at home and on non-work days^8,11^, and excessive increases in BP is considered to be long-term predictors of cardiovascular disorders^12–14^. However, how BP changes during the course of long working hours is unclear. In addition, underlying hemodynamic responses in increased BP, especially excessive increases in vascular responses, are also considered risk factors for cardiovascular disorders^12,15,16^. The underlying hemodynamic responses in increasing BP is that mean arterial pressure (MAP) is changed by responses of cardiac output (CO) and/or total peripheral resistance (TPR), and CO is changed by HR and/or stroke volume (SV). Our previous study^17^ investigated the hemodynamic responses of white-collar workers from 9:00 to 18:00 on a work day, and the results showed that the underlying hemodynamics of increasing MAP changed between the morning and the afternoon, although the MAP remained at the same level during these working hours. BP and HR from getting up to going to bed were also compared between 5 work days and 2 non-work days during a one-week period in the same study^17^. The results showed that BP significantly increased during working hours (from 9:00 to 18:00) and was higher than at the same time on non-work days. On the other hand, HR only changed between the morning and the afternoon on work days, suggesting that changes in the underling hemodynamics are due to the work but not circadian variation.

To recover from work-related fatigue, workers are permitted to take breaks at least a lunch break during working hours. According to the Labor Standards Law of Japan^18^, workers should not work more than 8 hours a day. A break longer than 45 minutes must be given if the working hours exceed 6 hours, and a break longer than 1 hour must be given if the working hours exceed 8 hours. In addition, the guideline regarding working with a visual display terminal also proposed a short break (10–15 minutes) between two 1-hour work periods^19^. However, these breaks mainly aim to reduce musculoskeletal and visual fatigue rather than the recovery of cardiovascular responses^20,21^. Few studies have examined whether these breaks provide the benefit of moderating cardiovascular responses in detail. A previous study^22^ reported that short breaks (3 minutes) relaxed the central nervous system but did not effectively moderate cardiovascular responses. Another study^23^ suggested that hemodynamic responses were not moderated within a 30-minute relaxation period after the task period. Whether long breaks, such as 45- to 60-minute breaks, moderate cardiovascular responses are unknown.

The aims of this study are to examine how long working hours influence hemodynamic responses, and to consider the necessity of long breaks under long working hours. We hypothesize that long working hours increase cardiovascular burdens and expect that long breaks benefit the moderation of these burdens. Since the work-related physiological response is not always conclusive regarding subjective fatigue^24^, we also examined subjective stress, fatigue, and sleepiness under long working hours in this study.

## Results

### Hemodynamic responses

**Figure 1:**
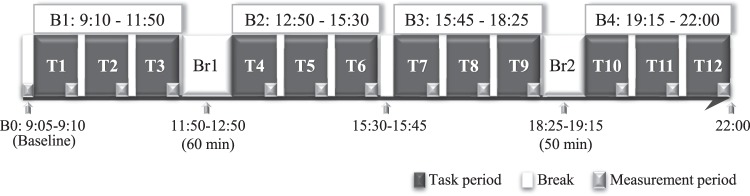
Time schedule of measurement. B: block; T: task period; Br: long break.

Thirty-eight male participants’ SBP were measured, as well as their diastolic blood pressure (DBP), MAP, SV, HR, CO, and TPR, at baseline from 9:05 to 9:10 and during task periods from 9:10 to 22:00 (Fig. 1).

**Figure 2:**
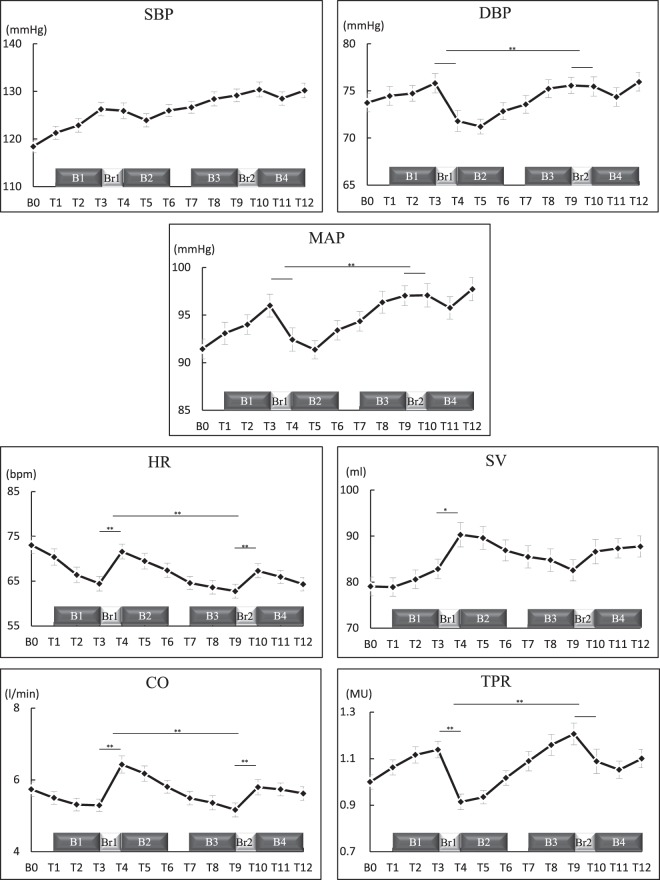
Hemodynamic responses exhibited during all measurement periods. SBP: systolic blood pressure; DBP: diastolic blood pressure; MAP: mean arterial pressure; HR: heart rate; SV: stroke volume; CO: cardiac output; TPR: total peripheral resistance; B: block; Br: long break; T: task period. Values are shown in mean and standard error. *p < 0.05; **p < 0.01.

The hemodynamic responses exhibited in all measurement periods are shown in Fig. 2. Results of the repeated one-way ANOVA showed that SBP (F (7.76, 286.97) = 15.68, p < 0.001, η_p_^2^ = 0.30, power = 1.00), DBP (F (7.55, 279.41) = 5.33, p < 0.001, η_p_^2^ = 0.13, power = 1.00), MAP (F (7.53, 278.61) = 8.79, p < 0.001, η_p_^2^ = 0.19, power = 1.00), HR (F (3.86, 142.69) = 29.29, p < 0.001, η_p_^2^ = 0.44, power = 1.00), SV (F (6.25, 231.16) = 10.60, p < 0.001, η_p_^2^ = 0.22, power = 1.00), CO (F (5.15, 190.44) = 15.74, p < 0.001, η_p_^2^ = 0.30, power = 1.00), and TPR (F (4.97, 183.92) = 14.01, p < 0.001, η_p_^2^ = 0.28, power = 1.00) significantly changed among measurement periods.

The results of multiple comparisons between baseline and task periods are shown in the upper part of Table 1. Compared to baseline, SBP and MAP increased, especially in the afternoon and at night; DBP did not significantly change throughout task periods. HR decreased in the morning, the latter half of afternoon, and at night; SV increased in the first half of afternoon and at night; CO decreased in the morning and during the last task period in the afternoon; and TPR increased in the morning and during the last task period in the afternoon.

**Table 1:** Results of the comparison between the baseline and task periods (n = 38).

	T1	T2	T3	T4	T5	T6	T7	T8	T9	T10	T11	T12
**Hemodynamic responses**												
SBP			*^a^	*^a^	*^a^	*^a^	*^a^	*^a^	*^a^	*^a^	*^a^	*^a^
DBP												
MAP								*^a^	*^a^	*^a^		*^a^
HR		*^b^	*^b^			*^b^	*^b^	*^b^	*^b^	*^b^	*^b^	*^b^
SV				*^a^	*^a^	*^a^				*^a^	*^a^	*^a^
CO		*^b^	*^b^						*^b^			
TPR	*^a^	*^a^	*^a^						*^a^			
**Subjective responses**												
Stress	*^a^		*^a^		*^a^	*^a^	*^a^	*^a^	*^a^	*^a^	*^a^	*^a^
Fatigue	*^a^	*^a^	*^a^	*^a^	*^a^	*^a^	*^a^	*^a^	*^a^	*^a^	*^a^	*^a^
Sleepiness						*^a^		*^a^				

SBP: systolic blood pressure; DBP: diastolic blood pressure; MAP: mean arterial pressure; HR: heart rate; SV: stroke volume; CO: cardiac output; TPR: total peripheral resistance; T: task period; *^a^significantly higher than baseline (p < 0.05); *^b^significantly lower than baseline (p < 0.05).

A comparison among task periods without long breaks is shown in Table 2. HR decreased but TPR increased in the morning. In the afternoon (T4 to T9), BP (SBP, DBP, and MAP) increased and cardiac responses (HR, SV, and CO) decreased, but vascular responses (TPR) increased during the task periods without long breaks. At night, HR decreased but other indices did not significantly change.

**Table 2:** Comparisons among task periods without long breaks.

	In the morning (T1 to T3)	In the afternoon (T4 to T9)	At night (T10 to T12)
SBP	ns	T5 < T9*	ns
DBP	ns	T4 to T5 < T9*; T5 < T8*	ns
MAP	ns	T4 < T9*; T5 < T7 to T9*; T6 < T8 to T9*	ns
HR	T1 > T2 to T3*	T4 to T5 > T6 to T9*; T6 > T7 to T9*; T7 > T9*	T10 > T12*
SV	ns	T4 to T5 > T9*	ns
CO	ns	T4 > T6 to T9*; T5 > T7 to T9*; T6 > T8 to T9*	ns
TPR	T1 < T3*	T4 to T5 < T6 to T9*; T6 < T8 to T9*	ns

SBP: systolic blood pressure; DBP: diastolic blood pressure; MAP: mean arterial pressure; HR: heart rate; SV: stroke volume; CO: cardiac output; TPR: total peripheral resistance; T: task period; *p < 0.05; ns: no significant difference.

A comparison between task periods before and after long breaks (Br1 and Br2) is shown in Fig. 2. The BP of T3 and T9 was not significantly different from T4 and T10, respectively. The HR of T3 was lower than T4, and T9 was lower than T10 (p < 0.05). The SV of T3 was lower than T4 (p < 0.05); the CO of T3 was lower than T4, and T9 was lower than T10 (p < 0.05). The TPR of T3 was higher than T4 (p < 0.05). Generally, cardiac responses increased, but vascular responses decreased after the long breaks, although BP did not significantly change after these breaks.

Comparisons of change values before and after long breaks (|T4-T3| vs. |T10-T9|) showed that for DBP, |T4-T3| > |T10-T9| (t (37) = 2.58, p < 0.05); for MAP, |T4-T3| > |T10-T9| (t (37) = 2.19, p < 0.05); for HR, |T4-T3| > |T10-T9| (t (37) = 3.12, p < 0.01); for SV, |T4-T3| did not significantly differ from |T10-T9| (t (37) = 1.76, p = 0.089); for CO, |T4-T3| > |T10-T9| (t (37) = 2.83, p < 0.01); and for TPR, |T4-T3| > |T10-T9| (t (37) = 2.33, p < 0.05). These results showed that, aside from SBP and SV, the change values of all the other indices became smaller in the evening than at noon.

### Subjective responses

The subjective stress (F (4.00, 147.87) = 16.30, p < 0.001, η_p_^2^ = 0.31, power = 1.00), fatigue (F (3.07, 113.70) = 38.66, p < 0.001, η_p_^2^ = 0.51, power = 1.00), and sleepiness (F (6.78, 250.00) = 2.24, p < 0.05, η_p_^2^ = 0.06, power = 0.82) were significantly different among measurement periods. Multiple comparisons between baseline and task periods are shown in the lower part of Table 1. Compared to baseline, stress increased in T1, T3, and T5 to T12 (p < 0.05); fatigue increased during all task periods (T1-T12, p < 0.05); and sleepiness increased in T6 and T8 (p < 0.05).

### Task performance

The total trials and correct rates of all tasks are shown in Table 3. The results of two-way ANOVAs showed that, for total trials, the main effect of the task (F (1.72, 63.66) = 8.35, p < 0.001, η_p_^2^ = 0.18, power = 0.93) was significant and that CW > MA > NC (p < 0.01). The main effect of the block (F (2.44, 90.12) = 2.03, p = 0.13, η_p_^2^ = 0.05, power = 0.45) and interactions between factors (F (4.40, 162.82) = 1.38, p = 0.24, η_p_^2^ = 0.04, power = 0.45) were not significant. For correct rates, the main effect of task (F (1.72, 63.66) = 8.35, p < 0.001, η_p_^2^ = 0.18, power = 0.93) was significant, and CW > MA and CW > NC (*p* < 0.05). The main effect of the block (F (2.44, 90.12) = 2.03, p = 0.13, η_p_^2^ = 0.05, power = 0.45) and interactions between factors (F (4.40, 162.82) = 1.38, p = 0.24, η_p_^2^ = 0.04, power = 0.45) were not significant.

**Table 3:** Total trials and correct rates (%) of all tasks (n = 38).

	Block 1	Block 2	Block 3	Block 4
**Total trials**				
Mental Arithmetic (MA)	437.03	464.39	462.26	468.95
SE	32.08	31.19	31.01	32.71
Color-word (CW)	788.74	758.97	804.29	818.97
SE	50.82	46.53	46.31	46.20
Number copy (NC)	267.74	281.39	284.71	292.71
SE	19.44	20.48	20.36	21.21
**Correct rates (%)**				
Mental Arithmetic (MA)	97.89	97.82	98.41	97.96
SE	0.25	0.27	0.21	0.28
Color-word (CW)	98.92	98.56	98.81	99.32
SE	0.23	0.47	0.43	0.17
Number copy (NC)	97.64	97.80	98.17	98.18
SE	0.37	0.40	0.35	0.33

Values are shown in mean and standard error (SE).

### Correlations

The results of the correlation analysis using average values across participants for each task period (n = 12) are shown in Table 4. SBP was significantly correlated with subjective stress (r = 0.83, p < 0.01) and fatigue (r = 0.89, p < 0.01) and was negatively correlated with HR (r = −0.59, p < 0.05). DBP was significantly correlated with TPR (r = 0.89, p < 0.01) and negatively correlated with HR (r = −0.71, p < 0.05) and CO (r = −0.79, p < 0.01). MAP was significantly correlated with TPR (r = 0.78, p < 0.01), subjective stress (r = 0.75, p < 0.01), and fatigue (r = 0.76, p < 0.01), but negatively correlated with HR (r = −0.59, p < 0.05). HR was significantly correlated with CO (r = 0.77, p < 0.01) but negatively correlated with TPR (r = −0.86, p < 0.01). SV was significantly correlated with CO (r = 0.80, p < 0.01) but negatively correlated with TPR (r = −0.65, p < 0.05). CO was negatively correlated with TPR (r = −0.95, p < 0.01). Subjective stress was significantly correlated with fatigue (r = 0.99, p < 0.01).

**Table 4:** Correlation coefficients between indices (n = 12).

	SBP	DBP	MAP	HR	SV	CO	TPR	Stress	Sleepiness	Fatigue
DBP	0.47									
MAP	0.81**	0.89**								
HR	−0.59*	−0.71*	−0.78**							
SV	0.42	−0.55	−0.15	0.24						
CO	−0.08	−0.79**	−0.58	0.77**	0.80**					
TPR	0.37	0.89**	0.78**	−0.86**	−0.65*	−0.95**				
Stress	0.83**	0.48	0.75**	−0.50	0.29	−0.12	0.32			
Sleepiness	0.20	0.25	0.29	−0.48	−0.05	−0.35	0.33	0.31		
Fatigue	0.89**	0.45	0.76**	−0.54	0.36	−0.10	0.32	0.99**	0.33	
CR	0.19	0.34	0.33	−0.01	−0.14	−0.09	0.18	0.39	−0.37	0.36

SBP: systolic blood pressure; DBP: diastolic blood pressure; MAP: mean arterial pressure; HR: heart rate; SV: stroke volume; CO: cardiac output; TPR: total peripheral resistance; CR: correct rate; *p < 0.05; **p < 0.01.

## Discussion

The results of this study support our hypothesis. Compared to baseline, SBP increased during all task periods, and MAP increased, especially in the latter half of these periods, although DBP did not significantly change during any task periods. Some previous studies reported that workers experienced a significant increase in SBP, which is considered to be a long-term predictor of incident hypertension^13,14,25^. It is known that BP increases in the early morning and reaches peak value between mid-morning and noon; it then falls progressively and drops to the minimum value during the night^26,27^. This study showed that SBP remained at an increased state throughout long working hours and that the response pattern was different from the circadian rhythms of BP, especially during the latter half of working hours. Additionally, SBP and MAP positively correlated with subjective stress and fatigue, and these results suggest that the increases in BP were due to the work-related strain but not the circadian rhythms. These results also suggest an increase in cardiovascular burden. Neither short breaks nor the long breaks showed significant effects on BP during long working hours.

Previous studies have reported that long working hours increased risks of cardiovascular diseases^3–5^, but other studies also reported a negative association between long working hours and cardiovascular diseases^28–31^. All of these studies were field investigations, and the different results could be mainly due to different study designs and different occupations of participants, as many occupational factors (e.g., work-related stress^32^, work contents and environment^33,34^, etc.) influence cardiovascular responses. This study, however, was performed in a controlled laboratory environment, and participants conducted the same tasks under the same time schedule. We believe that some biases of field investigation were avoided and that the results of this study are reliable and faithfully reflect the association between cardiovascular responses and long working hours, although it was designed as an acute exposure to long working hours, and the cumulative effects of long working hours on the cardiovascular system should also be verified in the future.

On average, BP is positively correlated with TPR but negatively correlated with HR. However, the underlying hemodynamics changed in a complicated manner during the working hours. Cardiac responses (HR, SV, and CO) decreased but vascular response (TPR) increased continually during task periods without long breaks. Previous studies have reported that cardiac responses are associated with physical activities and that decreases in HR during working hours were only detected in white-collar workers who sat for longer periods (66%) compared with blue-collar workers who sat for shorter periods (43%) at work^32,35^. The decreases in cardiac responses in this study were partly due to the long-term sedentary postures and low-level physical activities during continuous task periods without long breaks. In addition, a previous study^36^ has reported that prolonged monotonous daytime driving resulted in a linear decrease in HR. In this study, we repeatedly provided three slow-paced, simple mental tasks for a long time. For example, the previous study^23^ set the limitation at 3 seconds for a color-word (CW) task trial, but we set it at 10 seconds in this study, and the decreases in HR may also partly be due to the monotony of the tasks. The increased vascular response, however, could be mainly due to the accumulation of mental stress and fatigue during these continuous task periods because subjective stress and fatigue also increased during task periods. Previous studies suggested that excessive increases in vascular response increased the risks of cardiovascular diseases^12,15,16^. TPR showed significant and continuous increases, but cardiac response showed continuous decreases, especially during task periods in the afternoon, suggesting that long continuous work without long breaks may result in excessive cardiac and vascular responses. The short breaks (10 minutes), however, did not show obvious effects on cardiovascular responses, and these results agree with the previous study^23^.

On the other hand, the continuously decreased cardiac responses (HR, SV, and CO) increased, and the continuously increased vascular responses (TPR) decreased after the long breaks (of more than 50 minutes). The long breaks are considered to be effective in preventing excessive decreases in cardiac responses and increases in vascular responses. Differences between short and long breaks were the length of released time from task and intake of meals. Previous studies have reported that meal nutrition (such as carbohydrates, proteins, fats, etc.) and activities could influence cardiovascular responses^32,35,37,38^. Because the participants’ activities were not monitored and meals were not consistent among participants, we could not distinguish the effects among these factors, but we believe that the total effects of these factors resulted in the positive output. A comparison of the change values before and after Br1 and Br2 suggested that the effects of long breaks decreased in the evening. These results suggest that the effect of long breaks becomes weaker in the evening. Br2 was shorter than Br1 by 10 minutes but we do not believe this difference substantially affected the results because the cardiovascular responses did not qualitatively change during a short rest period^23,39^. The results of this study show that long breaks benefit workers by preventing these excessive responses especially excessive increases in TPR and we believe that taking more than one long break is crucial when long working hours cannot be avoided.

Subjective fatigue and stress increased during task periods, and sleepiness only increased during later afternoon (T6 and T8). There were no significant effects of breaks on these subjective indices, and these results suggested that the recovery of subjective fatigue and physiological responses may have different impacts. Task performance, however, was not different among task periods. In this study, we presented all tasks at a slow pace so that all participants could finish the trial within the limited time. We believe the slow pace of the task presentation resulted in all participants being able to finish the trials within the limited time, even when they were tired.

There are some limitations in this study. First, the experiment involved three simple tasks that are usually used in a laboratory, and the characteristics of these tasks may have influenced the hemodynamic responses. The results of this study should be verified in real workplaces because work in real workplaces is more complicated. Second, only male workers participated in the experiment; females should be included in future studies. Third, the influences of meals and activities during long breaks could not be distinguished in this study. We limited the meals to foods without much adipose and spice, but calories, carbohydrates, and adipose were not consistent. In addition, activities during breaks were not monitored or limited; all these elements need to be examined further to identify specific effect factors. Fourth, a previous study^40^ has reported that repeated measurements have a similar effect as a short break does because these measurements may cause changes in stressful situations. The repeated measurements in this study might be like additional breaks but we do not think these influences have an essential influence on the results because the measurement period was short and the response tendency within every block did not change significantly. Additionally, this study was a cross-sectional study; cumulative effects of long working hours are unknown, and follow-up investigation is necessary in the future. Finally, this experiment did not set a control condition, where the participants engaged in relaxing (non-work) activities during the entire experimental period. This fact did not allow us to make a more precise evaluation of the effects of breaks.

In conclusion, cardiovascular burden increased under long working hours. SBP increased throughout all working hours. Cardiac responses decreased but vascular responses increased continually during task periods without long breaks (of more than 50 minutes). The long breaks benefitted workers by preventing excessive hemodynamic responses, but the effects may become weaker with the extension of working hours. The results of this study suggest that taking more than one long break is crucial to reducing work-related cardiovascular burden when long working hours cannot be avoided.

## Methods

### Participants

Participants were recruited through a company that has a database of potential participants who registered as subjects before the study. The recruitment criteria included age (30–59 years), gender (men only), health status in general (individuals who had no previous history of cardiac disease, diabetes, asthma, cerebral stroke, chronic liver disorder, back problems, or mental disorders), and resting SBP and DBP in particular (resting SBP ≤ 140 mmHg, and resting DBP ≤ 90 mmHg). All potential participants who could join in this experiment were interviewed, and their resting BP was confirmed by a nurse using an arm-cuff digital BP monitor (CH-463E; Citizen Systems Japan Co., Ltd., Tokyo, Japan). All interviews started at 14:00 or 14:30 and ended by 16:00 or 16:30. As a result of the interviews, 39 healthy males participated, but one participant was excluded due to measurement errors. The mean age of the 38 remaining participants was 42.5 ± 8.5 years old, and all the participants had normal resting BP (SBP < 140 mmHg, and DBP < 90 mmHg). After the details of the study were explained, the participants practiced the experimental tasks and reserved a different measurement day. The participants were requested to refrain from exercise and alcohol intake and to sleep more than 6 hours before the measurement day.

All participants signed a written informed consent before the experiment. This study was approved by the Research Ethics Committee of the National Institute of Occupational Safety and Health of Japan (H2713), and all methods were performed in accordance with the relevant guidelines and regulations. The clinical trial registration number is UMIN000033103 (22/06/2018).

### Procedures

On each measurement day, only one participant came to the laboratory at approximately 8:30. After a 15-minute rest, the measurement sensors were worn on the middle finger of the non-dominant hand. The time schedule of measurement is shown in Fig. 1. Baseline data at rest were measured when the participant was sitting (B0: 9:05–9:10). PC-based tasks started at 9:10 and consisted of twelve 45-minute mental task periods (T1-T12), which were divided into 4 blocks. Block 1 was in the morning (B1: 9:10–11:50; T1 to T3), block 2 (B2: 12:50–15:30; T4 to T6) and block 3 (B3: 15:45–18:25; T7 to T9) were in the afternoon, and block 4 was at night (B4: 19:15–22:00; T10-T12). During task periods, the participant had to remain in a sedentary posture, and his behaviors were monitored in real time. During the last 5 minutes of each block, rest SBP and DBP were confirmed and if SBP > 180 mmHg or DBP > 110 mmHg, the experiment had to be stopped immediately. Eating and drinking were prohibited during these blocks.

After each task period, a 10-minute short break was provided within each block. In addition, two long breaks, a 1-hour break at noon (Br1: 11:50–12:50), and a 50-minute break in the evening (Br2: 18:25–19:15) were provided. During breaks, the participant stayed in an exclusive rest room with sofas and could drink water freely, and his behaviors were not limited or monitored. Meals could only be eaten during the long breaks and were limited to foods without much salt, adipose, and spices. Caffeinated beverages or smoking were prohibited throughout the measurement day.

### Mental tasks

The mental tasks included a CW task, a mental arithmetic (MA) task, and a number copy (NC) task. Each task was presented once per block. The presentation order was different among blocks, and counterbalance was considered among participants. In the CW task, a target word was presented on a computer screen. The target word was the name of a color (e.g., green), which was printed in a different color (e.g., yellow). Around the target word, six buttons labelled with the names of colors were presented. The participants were instructed to press the button corresponding to the print color of the target word within 10 seconds. In the case of the example, the correct reaction would be to press the button labelled “yellow.” The MA task was an addition task. Two random 2-digit numbers (10–49) were presented on a computer screen, and the participants had to add them mentally and type the result within 20 seconds using a 10-key pad. In the NC task, a random 10-digit number was presented on a computer screen, and the participants had to type the same number within 20 seconds using a 10-key pad. If incorrect result was detected or the response time exceeded the limitation, an alarm sounded. Total trials and correct answers were recorded automatically for all tasks.

### Measurement indices and analysis

As hemodynamic response indices, SBP, DBP, MAP, CO, HR, SV, and TPR were measured using a noninvasive, continuous hemodynamic monitor (Finapres Pro, Finapres Medical Systems, Inc., Netherlands). The hemodynamic indices were measured for 5 minutes at the end of each task period, and sensors were removed immediately after measurement. After the measurement, subjective fatigue, stress, and sleepiness were measured using the visual analogue scale. Repeated one-way ANOVAs were conducted to examine differences in physiological and subjective responses among measurement periods. Repeated two-way ANOVAs (3 Tasks × 4 Blocks) were conducted to examine task performance. Multiple comparisons (Bonferroni) were conducted to further examine the significant results. If the result of Mauchly’s sphericity test was significant, Greenhouse-Geisser correction was used to estimate epsilon to correct the degree of freedom of the F value. Measures of effect size (η_p_^2^) and power were also reported. To compare the changes before and after long breaks (Br1 and Br2), absolute values of changes (|T4-T3| vs |T10-T9|) were compared using paired t-tests. A correlation analysis between BP and the other indices was also conducted using the average value across the participants for each task period. The level of significance was set at 0.05. Statistical analyses were carried out using IBM SPSS Statistics 19 (IBM Corp., Armonk, NY, USA).

### Ethics approval

All participants signed a written informed consent before the experiment. This study was approved by the Research Ethics Committee of the National Institute of Occupational Safety and Health of Japan (H2713) and all methods were performed in accordance with the relevant guidelines and regulations.
